# Severity of experimental traumatic brain injury modulates changes in concentrations of cerebral free amino acids

**DOI:** 10.1111/jcmm.12998

**Published:** 2016-10-03

**Authors:** Angela Maria Amorini, Giacomo Lazzarino, Valentina Di Pietro, Stefano Signoretti, Giuseppe Lazzarino, Antonio Belli, Barbara Tavazzi

**Affiliations:** ^1^ Institute of Biochemistry and Clinical BiochemistryCatholic University of RomeRomeItaly; ^2^ Neuroscience and Ophthalmology groupSchool of Clinical and Experimental MedicineCollege of Medical and Dental SciencesUniversity of BirminghamBirminghamUK; ^3^ Division of NeurosurgeryDepartment of Neurosciences Head and Neck SurgeryS. Camillo HospitalRomeItaly; ^4^ Division of Medical BiochemistryDepartment of Biomedical and Biotechnological SciencesUniversity of CataniaCataniaItaly; ^5^ National Institute for Health Research Surgical Reconstruction and Microbiology Research CentreQueen Elizabeth HospitalBirminghamUK

**Keywords:** cerebral free amino acids, excitotoxicity, high performance liquid chromatography, methyl‐cycle, *N*‐acetylaspartate, mild traumatic brain injury, severe traumatic brain injury

## Abstract

In this study, concentrations of free amino acids (FAA) and amino group containing compounds (AGCC) following graded diffuse traumatic brain injury (mild TBI, mTBI; severe TBI, sTBI) were evaluated. After 6, 12, 24, 48 and 120 hr aspartate (Asp), glutamate (Glu), asparagine (Asn), serine (Ser), glutamine (Gln), histidine (His), glycine (Gly), threonine (Thr), citrulline (Cit), arginine (Arg), alanine (Ala), taurine (Tau), γ‐aminobutyrate (GABA), tyrosine (Tyr), S‐adenosylhomocysteine (SAH), l‐cystathionine (l‐Cystat), valine (Val), methionine (Met), tryptophane (Trp), phenylalanine (Phe), isoleucine (Ile), leucine (Leu), ornithine (Orn), lysine (Lys), plus *N*‐acetylaspartate (NAA) were determined in whole brain extracts (*n* = 6 rats at each time for both TBI levels). Sham‐operated animals (*n* = 6) were used as controls. Results demonstrated that mTBI caused modest, transient changes in NAA, Asp, GABA, Gly, Arg. Following sTBI, animals showed profound, long‐lasting modifications of Glu, Gln, NAA, Asp, GABA, Ser, Gly, Ala, Arg, Citr, Tau, Met, SAH, l‐Cystat, Tyr and Phe. Increase in Glu and Gln, depletion of NAA and Asp increase, suggested a link between NAA hydrolysis and excitotoxicity after sTBI. Additionally, sTBI rats showed net imbalances of the Glu‐Gln/GABA cycle between neurons and astrocytes, and of the methyl‐cycle (demonstrated by decrease in Met, and increase in SAH and l‐Cystat), throughout the post‐injury period. Besides evidencing new potential targets for novel pharmacological treatments, these results suggest that the force acting on the brain tissue at the time of the impact is the main determinant of the reactions ignited and involving amino acid metabolism.

## Introduction

Under physiological conditions, free amino acids (FAA) and amino group containing compounds (AGCC), exert fundamental biochemical roles in numerous brain functions. They can roughly be divided into different categories: (1) those directly involved in neurotransmission, such as glutamate (Glu) and γ‐aminobutyrric acid (GABA) 1; (2) those participating to neurotransmission, such as d‐serine (d‐Ser) and glycine (Gly) 2; (3) those indirectly controlling neurotransmission, such as glutamine (Gln), aspartate (Asp), triptophane (Trp), phenylalanine (Phe) and tyrosine (Tyr) 3, 4, 5; (4) those indirectly involved in cellular energy metabolism, such as leucine (Leu), isoleucine (Ile) and valine (Val) 6; (5) those involved in specific metabolic pathways, such as methione (Met), cystathionine (l‐Cystat) and *S*‐adenosylhomocysteine (SAH) 7.

Besides, several of them have multiple functions: for instance, Glu and Gly are both substrates necessary for the biosynthesis of reduced glutathione (GSH), the main cerebral low molecular weight antioxidant 8; Glu is also needed for the synthesis of an additional neurotransmitter, namely *N*‐acetylaspartylglutamate (NAAG) 9; Asp is one of the substrate used for the synthesis of *N*‐acetylaspartate (NAA), the most abundant, neuron specific, *N*‐acetylated amino acid found in the brain 10; both Asp and Glu participate in the regulation of brain energy metabolism as anaplerotic and cataplerotic compounds of the tricarboxylic acid (TCA) cycle 11.

According to data from animal studies, it appears that the physiological pool of FAA within whole brain tissue is not subjected to significant fluctuations 12, 13, whereas the levels of these compounds are affected, to various degrees, in some cerebral pathologies. Experimental brain ischaemia and reperfusion 14, animal model of amyotrophic lateral sclerosis 15 and experimental seizures 16 have been shown to influence several of the aforementioned substances.

Traumatic brain injury (TBI) is the leading cause of death and disability in young adults (below 45 years of age) in Western countries 17. Excluding prevention, currently there are no therapies available to reduce TBI‐associated damage, partly owing to our incomplete understanding of the cellular and molecular mechanisms of injury 18, so that it is not incorrect to state that TBI patients are still waiting for a satisfactory pharmacological approach. As thoroughly scrutinized and demonstrated, given the visible, morphological damage, a further, profound, hidden and somehow ‘invisible’ biochemical harm also exists, initiated by the inertial forces acting on the brain tissue at the time of impact (rotational, translational). These forces trigger a cascade of molecular events altering a plethora of cellular functions including ionic homeostasis 19, mitochondrial functions 20, energy metabolism 21, neurotransmission 22, signal transduction 23, protein folding 24, gene expression 25, apoptosis 26. Various experimental studies have ultimately demonstrated that these and other changes are strictly related to severity of injury 27, on turn related to the amount of the mechanical energy transferred at the time of impact, with mild TBI (mTBI) mostly causing spontaneously resolving changes 28, and with severe TBI (sTBI) mainly provoking permanent alterations of pivotal cerebral cell functions 29, 30.

Notwithstanding the impressive, constantly increasing, number of studies on TBI, up to date there are no comprehensive data on changes potentially occurring to all FAA and ACCG following TBI. Most of the studies have so far focused on amino acids mainly involved in neurotransmission 31, 32, 33. None has been carried out in TBI of different severity. Hence, very little is known in relation to about 10–15 compounds involved in various cell functions and how such a pathophysiology highlight might contribute to potential new targets for pharmacological interventions.

In this study, by using the same brain samples deriving from cohorts of control rodents and of rats experiencing mTBI or sTBI, previously used to assess various biochemical and molecular effects caused by graded TBI 34, 35, 36, we have determined the concentrations of FAA and ACCG at different times following the two levels (mild and severe) of experimental diffuse closed head injury.

## Materials and methods

### Chemicals

High performance liquid chromatography (HPLC)‐grade tetrabutylammonium hydroxide, used as the pairing reagent in the separation of *N*‐acetylaspartate, (NAA), ortophtalaldehyde (OPA) and 3‐mercaptopropionic acid (MPA), used as the reagents to obtain fluorescent‐derivatized amino compounds, as well as ultrapure NAA, standard and non‐standard amino acids and amino compounds, were supplied from Sigma‐Aldrich (St. Louis, MO, USA). HPLC‐grade acetonitrile, methanol and tetrahydrofurane were purchased from J.T Baker (Mallinckrodt Baker, Deventer, the Netherlands). All other chemicals were of the highest purity available from commercial sources.

### Experimental protocol

The experimental protocol was approved by the Ethical Committee of the Catholic University of Rome, according to international standards and guidelines for animal care. As previously described, Male Wistar rats of 300–350 g were randomly divided into three groups: (1) sham‐operated as control; (2) mild diffuse TBI (mTBI group); (3) severe diffuse TBI (sTBI group) 34, 35, 36. As for the anaesthetic mixture, animals received 35 mg/kg b.w. ketamine and 0.25 mg/kg b.w. midazolam by i.p. injection. Since we were interested in a type of trauma producing a diffuse axonal injury, graded TBI (mTBI or sTBI) was induced according to the weight drop impact acceleration model characterized by causing diffuse axonal damage 37. After anaesthesia, a metal disk was fixed onto the central portion of the skull, between the coronal and lambdoid sutures, to prevent skull fracture and to homogenously distribute the force acting at the time of impact. Mild or severe TBI were induced by dropping a cumulative weight of 450 g from 1 or 2 m height and knowing to cause, respectively, a mTBI or a sTBI either histopathologically or biochemically 34, 35, 36, 37, 38. At 6, 12, 24, 48 and 120 hrs from injury, rats were again anaesthetized (*n* = 6 for each time‐point in both groups of injured animals) and then immediately killed. Sham‐operated animals were killed 120 hr after the initial anaesthesia (*n* = 6) and used as controls.

### Tissue preparation for the determination of amino acids, amino‐compounds and NAA

As described elsewhere 38, an *in vivo* craniectomy was performed in all animals during anaesthesia, the brain was exposed, sharply cut along the sagittal fissure and the two hemispheres were freeze‐clamped in liquid nitrogen to minimize metabolite loss 34, 35, 36.

The tissue preparation was performed on one hemisphere, using the organic solvent deproteinizing procedure described elsewhere 39. A total of three right + three left hemispheres for each time‐point in both groups of rats (mTBI and sTBI), as well as for the control group, were processed as described above to give clear aqueous 10% protein‐free tissue extracts suitable for the HPLC analysis of free amino acids, amino‐compounds and NAA.

### HPLC separation of amino acids, amino‐compounds and NAA

The HPLC apparatus (ThermoFisher Italia, Rodano, Milan, Italy) consisted of a P4000 quaternary pump system, an AS3000 autosampler and a highly sensitive UV6000LP photodiode array detector equipped with a 5 cm light path flow cell and set up between 200 and 400 nm wavelength. Data acquisition and analysis were performed by a PC using the ChromQuest^®^ software package provided by the HPLC manufacturer.

The simultaneous determination of 24 primary FAA and ACCG, including Asp, Glu, asparagine (Asn), serine (Ser), Gln, histidine (His), Gly, threonine (Thr), citrulline (Cit), arginine (Arg), alanine (Ala), taurine (Tau), GABA, Tyr, SAH, l‐Cystat, Val, Met, Trp, Phe, Ile, Leu, ornithine (Orn) and lysine (Lys), plus the internal standard norvaline (Norval), was performed using the pre‐column derivatization of the sample with a mixture of OPA and MPA, as described elsewhere 40. Briefly, the derivatization mixture composed by 25 mmol/l OPA, 1% MPA, 237.5 mmol/l sodium borate, pH 9.8 was prepared daily and placed in the autosampler. The automated precolumn derivatization of the samples (15 μl) with OPA‐MPA was carried out at 24°C and 25 μl of the derivatized mixture were loaded onto the HPLC column (Hypersil C‐18; 250 × 4.6 mm, 5 μm particle size, thermostated at 21°C) for the subsequent chromatographic separation. In the case of Glu, deproteinized brain extracts were diluted 20 times with HPLC‐grade H_2_O prior to the derivatization procedure and subsequent injection. Separation of OPA‐AA and OPA‐ACCG was carried out at a flow rate of 1.2 ml/min using two mobile phases (mobile phase A = 24 mmol/l CH_3_COONa + 24 mmol/l Na_2_HPO_4_ + 1% tetrahydrofurane + 0.1% trifluoroacetic acid, pH 6.5; mobile phase B = 40% CH_3_OH + 30% CH_3_CN + 30% H_2_O), using an appropriate step gradient 41. Assignment and calculation of the OPA‐AA and OPA‐ACCG in chromatographic runs of whole brain extracts were carried out at 338 nm wavelengths by comparing retention times and areas of peaks with those of peaks of chromatographic runs of freshly prepared ultra‐pure standard mixtures with known concentrations.

The determination of NAA was carried out isocratically on 10 μl of deproteinized whole brain extracts, as described elsewhere 42. Assignment and calculation of NAA was carried out at 206 nm wavelengths by comparing retention time, absorption spectrum and area of the NAA peak in sample runs with those of chromatographic runs of freshly prepared ultra‐pure NAA with known concentration.

### Statistical analysis

Normal data distribution was tested using the Kolmogorov‐Smirnov test. The within group comparison at each time was performed by the one‐way analysis of variance (anova). Differences across groups were estimated by the two‐way anova for repeated measures. Fisher's protected least square was used as the *post hoc* test. Only two‐tailed *P*‐values of <0.05 were considered statistically significant.

## Results

**Figure 1:**
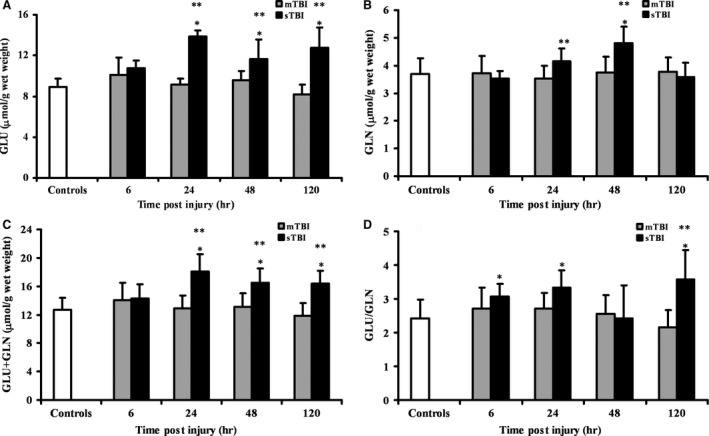
Time course changes of cerebral concentrations of Glu (**A**), Gln (**B**), Glu + Gln (**C**) and Glu/Gln ratio (**D**) determined by HPLC in deproteinized tissue extracts of rats receiving mTBI or sTBI. Controls are represented by a group of sham operated rats (*n* = 6). Values at each time‐point are the mean of six animals (3 left + 3 right hemispheres). Standard deviations are represented by vertical bars. Tissue preparation, sample processing, pre‐column derivatization with OPA, and HPLC conditions for the separation of OPA‐amino acid adducts are fully described under Materials and Methods. *Significantly different from controls, *P* < 0.01. **Significantly different from corresponding time of mTBI rats, *P* < 0.05.

In Fig. 1, the changes in Glu, Gln, Glu + Gln and Glu/Gln ratio at different times post‐graded TBI are illustrated. Concentration of whole brain Glu after mTBI (Fig. 1A) show a slight but not significant increase at 6, 24 and 48 hrs post impact (+12%, +2% and +7%, respectively). Differently, in animal receiving sTBI, Glu values were significantly higher than controls at 24 (+55%), 48 (+30%) and 120 hrs (+43%) after insult (*P* < 0.01 with respect to control values). Concentration of Glu at these time‐points was also significantly higher than that recorded in mTBI rats (*P* < 0.01). The determination of Gln (Fig. 1B) showed that mTBI did not affect the concentration of this amino acid, whilst an increase at 24 and 48 hrs post‐sTBI was recorded (maximal increase = +30% at 48 hrs; *P* < 0.01 with respect to both controls and mTBI rats). In consequence of the differential changes of Glu and Gln in the two types of trauma, the Glu + Gln sum (Fig. 1C) was not affected by mTBI, whilst higher values were recorded at 24, 48 and 120 hrs after sTBI (+43%, +30% and +29%, respectively; *P* < 0.01 compared to both controls and mTBI rats). Also the Glu/Gln ratio (Fig. 1D) did not increase following mTBI and underwent significant increase in sTBI rats at 6, 24 and 120 hrs post‐injury (+26%, +39% and +48%, respectivley, *P* < 0.01 compared to control rats).

**Figure 2:**
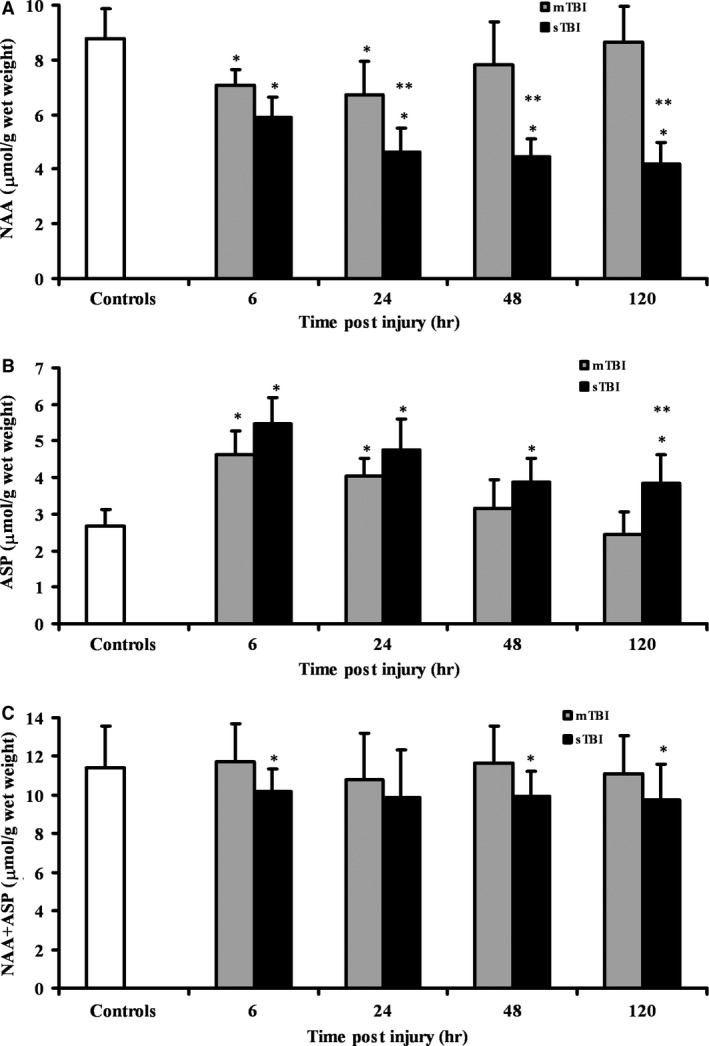
Time course changes of cerebral concentrations of NAA (**A**), Asp (**B**), and NAA + Asp (**C**) determined by HPLC in deproteinized tissue extracts of rats receiving mTBI or sTBI. Controls are represented by a group of sham operated rats (*n* = 6). Values at each time‐point are the mean of six animals (3 left + 3 right hemispheres). Standard deviations are represented by vertical bars. Tissue preparation, sample processing, pre‐column derivatization with OPA, and HPLC conditions for the separation of NAA and of OPA‐amino acid adducts are fully described under Materials and Methods. *Significantly different from controls, *P* < 0.01. **Significantly different from corresponding time of mTBI rats, *P* < 0.01.

Because of the possible interdependence between NAA and Asp variations, in Fig. 2 we compared the changes in NAA, Asp and NAA + Asp in whole brain extracts of mTBI and sTBI rats at different times post‐injury. Variations of NAA (Fig. 2A) confirmed previous observations 34, 35, 36 showing a transient decrease at 6 and 24 hrs (−19% and −23%, respectively; *P* < 0.01 compared to controls) and a full restoration at 48 and 120 hrs after mTBI, and a steady profound depletion at any time after sTBI (−33%, −47%, −49% and −52% at 6, 24, 48 and 120 hrs, respectively; *P* < 0.001 compared to both controls and mTBI rats). As illustrated in Fig. 2B, when considering the change in brain Asp concentration (calculated in μmol/g wet weight), an increase in 1.96 and 1.36 μmol/g wet weight was recorded at 6 and 24 hrs, respectively, after mTBI (*P* < 0.01 compared to controls). Concentration of Asp in sTBI animals increased by 2.80, 2.07, 1.19 and 1.18 μmol/g wet weight at 6, 24, 48 and 120 hrs, respectively (*P* < 0.01 compared to both controls and mTBI rats). According to the data of Fig. 2C, the increase in Asp observed in mTBI rats equalled (in concentration) the decrease in NAA, so that the sum of these two interconnected compounds (NAA + Asp) did not change at any time after a mild injury. The NAA + Asp sum in brain tissue of sTBI animals was significantly lower than controls at 6 (−11%; *P* < 0.05), 48 (−13%; *P* < 0.05) and 120 hrs post‐injury (−15%; *P* < 0.05), indicating that the increase in Asp (in concentration) was not equal to the decrease in NAA (in concentration), i.e. Asp produced by NAA hydrolysis was converted into different compounds or was cleared out of the cerebral tissue.

**Figure 3:**
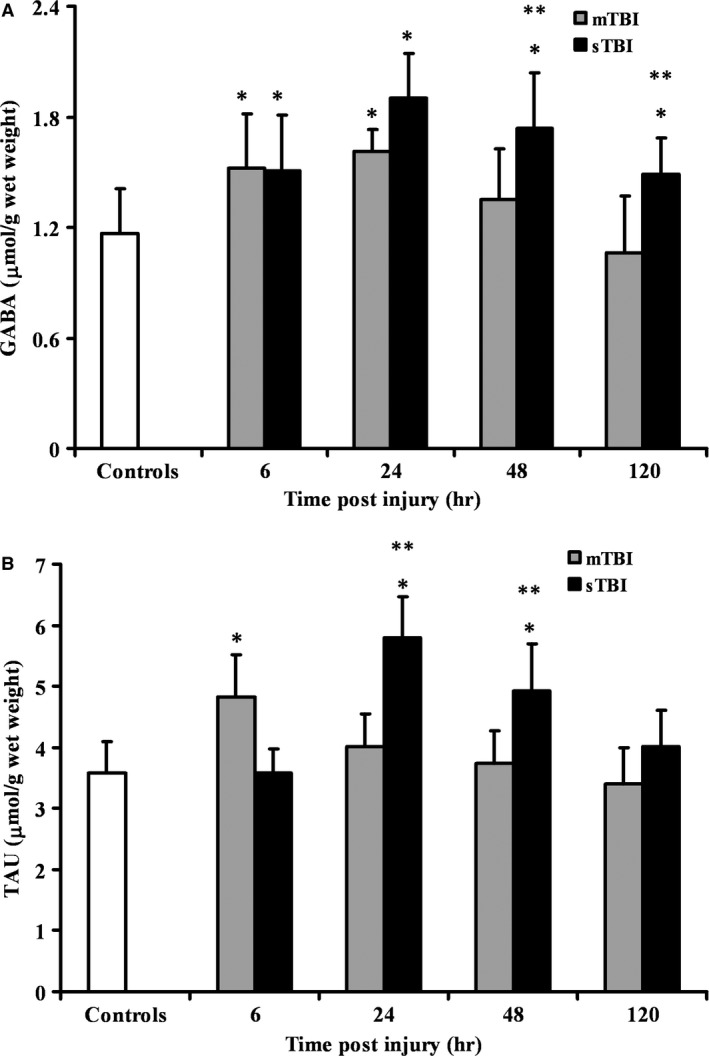
Time course changes of cerebral concentrations of GABA (**A**), and Tau (**B**) determined by HPLC in deproteinized tissue extracts of rats receiving mTBI or sTBI. Controls are represented by a group of sham operated rats (*n* = 6). Values at each time‐point are the mean of six animals (3 left + 3 right hemispheres). Standard deviations are represented by vertical bars. Tissue preparation, sample processing, pre‐column derivatization with OPA, and HPLC conditions for the separation of OPA‐amino acid adducts are fully described under Materials and Methods. *significantly different from controls, *P* < 0.01. **significantly different from corresponding time of mTBI rats, *P* < 0.01.

The influence of graded TBI on the main inhibitory neurotransmitter amino acid (GABA) and one important amino compound osmolite (Tau) are shown in Fig. 3. Animals receiving mTBI showed a significant increase in GABA (Fig. 3A) at 6 and 24 hrs post‐impact (+30% and +38%; *P* < 0.01 respect to controls), with values not significantly different from those of controls at 48 and 120 hrs after insult. Severe injury caused an increase in GABA at any time post‐impact (+29%, +62%, +32% and +27%, respectively, at 6, 24, 48 and 120 hrs; *P* < 0.01 compared to controls and mTBI rats). In mTBI injured rats, Tau increased only after 6 hrs from the insult (+35%, *P* < 0.01 respect to controls), whilst in sTBI injured rats an increase in Tau was recorded at 24 and 48 hrs post‐impact (+62% and +38%, respectively, *P* < 0.01 compared to controls and mTBI rats).

**Figure 4:**
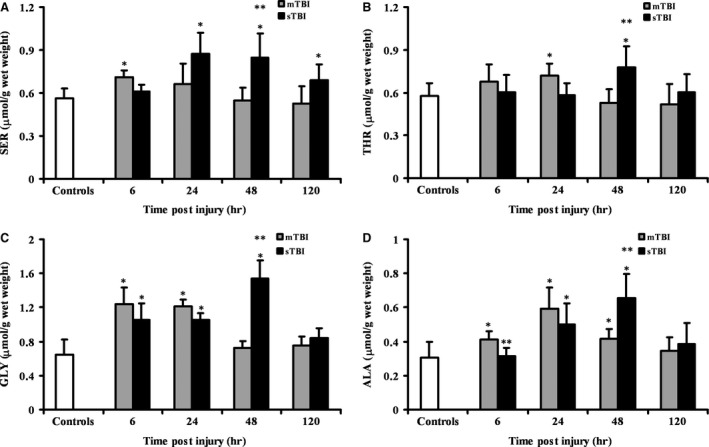
Time course changes of cerebral concentrations of Ser (**A**), Thr (**B**), Gly (**C**) and Ala (**D**) determined by HPLC in deproteinized tissue extracts of rats receiving mTBI or sTBI. Controls are represented by a group of sham operated rats (*n* = 6). Values at each time‐point are the mean of six animals (3 left + 3 right hemispheres). Standard deviations are represented by vertical bars. Tissue preparation, sample processing, pre‐column derivatization with OPA, and HPLC conditions for the separation of OPA‐amino acid adducts are fully described under Materials and Methods. *Significantly different from controls, *P* < 0.05. **Significantly different from corresponding time of mTBI rats, *P* < 0.01.

Ser, Thr, Gly and Ala were differently affected by the two grades of TBI. As shown in Fig. 4A–D, mTBI provoked sporadic, modest changes in these four amino acids, mainly within the first 24 hrs post‐trauma. More evident and prolonged changes occurred after sTBI, particularly for Ser, Gly and Ala all showing identical time courses with maximal increases recorded at 48 hrs post‐insult.

**Figure 5:**
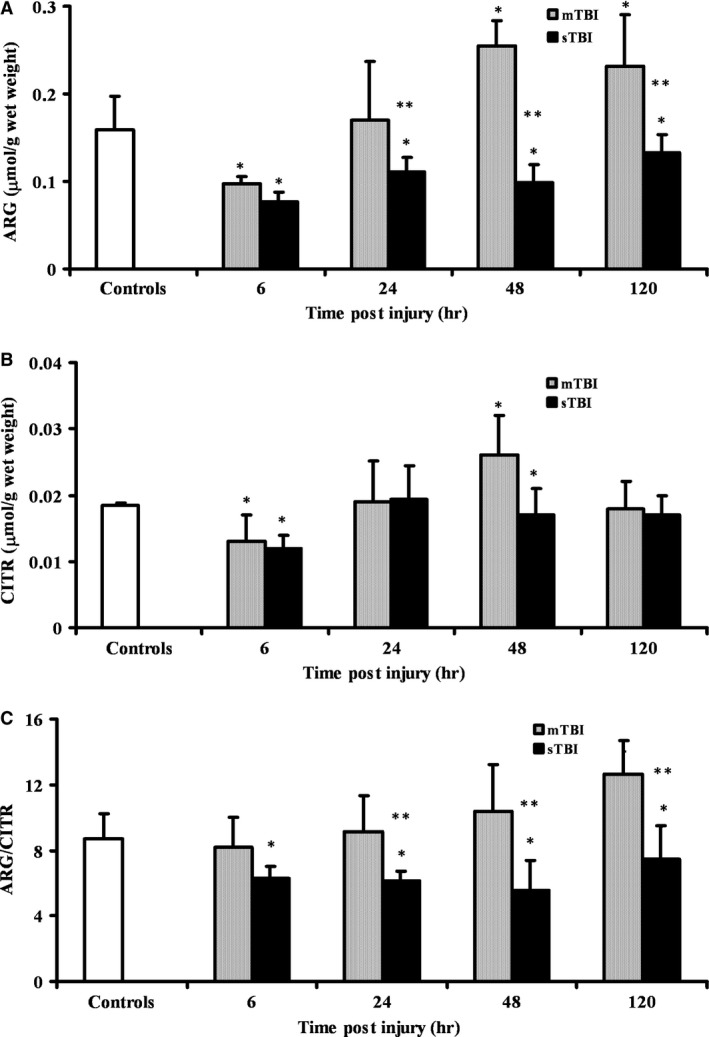
Time course changes of cerebral concentrations of Arg (**A**), Citr (**B**), and Arg/Citr ratio (**C**) determined by HPLC in deproteinized tissue extracts of rats receiving mTBI or sTBI. Controls are represented by a group of sham operated rats (*n* = 6). Values at each time‐point are the mean of six animals (3 left + 3 right hemispheres). Standard deviations are represented by vertical bars. Tissue preparation, sample processing, pre‐column derivatization with OPA, and HPLC conditions for the separation of OPA‐amino acid adducts are fully described under Materials and Methods. *Significantly different from controls, *P* < 0.01. **Significantly different from corresponding time of mTBI rats, *P* < 0.01.

Figure 5 illustrates changes in the concentrations of Arg and Citr, and in the Arg/Citr ratio after mTBI or sTBI. Following a mild injury, Arg initially decreased (−40% at 6 hrs, *P* < 0.01 compared to controls) and then increased resulting +62% and +48% higher than controls (*P* < 0.01) at 48 and 120 hrs, respectively. Consistently lower values of Arg than controls were found in brain homogenates of sTBI animals (−52%, −30%, −38%, −16% at 6, 24, 48, and 120 hrs, respectively; *P* < 0.01 compared to both controls and mTBI rats). In mTBI rats, Citr underwent a −28% reduction at 6 hrs (*P* < 0.01), followed by a recovery at 24 hrs, an increase by 44% at 48 hrs (*P* < 0.01) and a further normalization at 120 hrs post‐trauma. In sTBI animals, a −33% in Citr concentration was detected at 6 hrs, followed hereinafter by normalization to pre‐impact values. As a consequence of the differential Arg and Citr changes occurring in the two levels of injury, the Arg/Citr ratio, which is an indirect indicator of nitric oxide (NO) production by endothelial, neuronal and inducible nitric oxide synthases (eNOS, nNOS and iNOS), was unchanged at 6, 24 and 48 hrs and was increased by 45% at 120 hrs (*P* < 0.01) after mTBI, whilst it was consistently lower at any time post‐sTBI (*P* < 0.01 compared to both control and mTBI rats).

The variations of the remaining FAA and ACCG, determined at different times post‐mTBI or ‐sTBI in deproteinized brain homogenates, are summarized in Table 1. None of the 10 FAA (Asn, His, Tyr, Val, Met Trp, Phe, Ile, Leu Lys) and of the two ACCG (SAH and l‐Cystat) were affected by a mild level of TBI. Conversely, the amino acids (Met and l‐Cystat and the amino group containing compound (SAH) involved in the so called methyl‐cycle, which regulates numerous methylation reactions within a living cell, underwent remarkable changes, with significant increase in SAH and concomitant decrease in Met and increase in l‐Cystat) particularly evident between 24 and 120 hrs post‐injury (*P* < 0.01 compared to both control and mTBI rats).

**Table 1:** Time course of changes of amino acids and amino group containing compounds determined in deproteinized brain homogenates of rats receiving mTBI or sTBI

Time post‐injury (hours)	ASN	HIS	TYR	SAH	VAL	MET	TRP	PHE	ILE	LEU	LYS	l‐Cystat
Controls												
	0.108 (0.035)	0.045 (0.011)	0.123 (0.047)	0.026 (0.010)	0.049 (0.005)	0.015 (0.004)	0.013 (0.003)	0.023 (0.003)	0.030 (0.010)	0.015 (0.005)	0.206 (0.042)	0.147 (0.031)
mTBI												
6	0.132 (0.009)	0.052 (0.007)	0.143 (0.025)	0.037 (0.014)	0.048 (0.007)	0.013 (0.003)	0.013 (0.003)	0.031 (0.008)	0.029 (0.008)	0.013 (0.003)	0.243 (0.072)	0.185 (0.046)
24	0.101 (0.012)	0.039 (0.003)	0.122 (0.021)	0.045^a^ (0.011)	0.036 (0.017)	0.014 (0.004)	0.012 (0.002)	0.022 (0.002)	0.027 (0.005)	0.013 (0.007)	0.185 (0.034)	0.191 (0.039)
48	0.106 (0.018)	0.061 (0.009)	0.131 (0.023)	0.032 (0.009)	0.040 (0.011)	0.017 (0.005)	0.014 (0.004)	0.031 (0.011)	0.031 (0.005)	0.018 (0.006)	0.192 (0.023)	0.173 (0.035)
120	0.089 (0.019)	0.049 (0.016)	0.116 (0.037)	0.028 (0.005)	0.039 (0.006)	0.019 (0.002)	0.010 (0.004)	0.025 (0.005)	0.032 (0.007)	0.016 (0.002)	0.189 (0.056)	0.147 (0.038)
sTBI												
6	0.125 (0.017)	0.054 (0.013)	0.111 (0.028)	0.064^a^^,^ b (0.024)	0.043 (0.019)	0.009^a^^,^ b (0.004)	0.013 (0.002)	0.031 (0.008	0.026 (0.007)	0.0011 (0.006)	0.265 (0.064)	0.200^a^ (0.025)
24	0.134 (0.050)	0.046 (0.031)	0.166^a^ (0.038)	0.077^a^ (0.050)	0.038 (0.004)	0.010^a^^,^ b (0.002)	0.016^a^ (0.004)	0.049^a^^,^ b (0.003)	0.029 (0.013)	0.011 (0.004)	0.230 (0.078)	0.279^a^^,^ b (0.062)
48	0.116 (0.048)	0.060 (0.029)	0.159 (0.074)	0.077^a^^,^ b (0.021)	0.057 (0.018)	0.011^a^^,^ b (0.003)	0.023^a^^,^ b (0.006)	0.046^a^^,^ b (0.022)	0.043 (0.020)	0.014 (0.003)	0.202 (0.055)	0.337^a^^,^ b (0.030)
120	0.091 (0.028)	0.046 (0.008)	0.123 (0.034)	0.043^a^^,^ b (0.006)	0.042 (0.014)	0.010^a^^,^ b (0.001)	0.012 (0.005)	0.033 (0.008)	0.038^a^ (0.010)	0.014 (0.005)	0.190 (0.049)	0.202^a^^,^ b (0.058)

Each value is the mean (S.D.) of six different brain samples (3 left + 3 right hemispheres) and is expressed as μmol/g wet weight.

a Significantly different from controls, *P* < 0.01.

b Significantly different from corresponding times of mTBI rats, *P* < 0.01.

## Discussion

In view of their multiple and complex biochemical roles, FAA and ACCG are of crucial importance for maintaining brain tissue metabolic homeostasis 6, 7. For this reason, several studies have been carried out in both acute and chronic cerebral pathologies, in the majority of cases focussing on changes occurring to amino acidic neurotransmitters 4, 11. TBI studies have also mainly focused on amino acids involved in neurotransmission 33, especially with regard to changes in their extracellular concentrations 32, 43.

Results reported in the present study provide a comprehensive picture of the effects of two levels of TBI, characterized by diffuse axonal injury, on the cerebral concentrations of 18 standard FAA and 6 ACCG (including two amino acids of the urea cycle), and of the main neuron specific *N*‐acetylated amino acid (NAA). A general remark is that, once again, mTBI modestly and transiently affects the concentrations of FAA and ACCG, as well as of NAA and it is clearly distinguishable from sTBI causing a profound, long‐lasting imbalance in the homeostasis of the aforementioned substances.

Data referring to Glu and Gln levels indicate that mTBI did not cause significant excitotoxic conditions, possibly maintaining unaltered the Glu‐Gln cycle between neurons and astrocytes 11. Conversely, sTBI induced a net increase in both Glu and Gln throughout the observational period (120 hrs), with high concentrations of Glu any time post‐impact. This indicates that with this level of injury Glu excitotoxicity might be caused by a combined increase in extracellular Glu 32, 43, 44 and in its total cerebral concentration. Moreover, the increase in the Glu/Gln ratio following sTBI should indicate an impairment in the neuron‐astrocyte Glu‐Gln cycle which should contribute to a decreased rate in Glu removal from the extracellular milieu and consequent exacerbation of the cytotoxic effects of excess Glu outside cells 11, 33.

It can be hypothesized that the Glu increase is related to NAA changes. As previously observed, alterations in NAA homeostasis are transient after mTBI and permanent after sTBI 45. These are caused by biochemical and molecular changes occurring to mitochondrial functions, as well as to the expression of the genes encoding for the enzymes catalysing NAA biosynthesis and degradation 35. In the present study, we could show that only after sTBI the decrease in NAA is not stoichiometrically accompanied by an in increase in Asp (Fig. 2). By subtracting the pre‐impact NAA concentration, it is possible to calculate that 2.87, 4.12, 4.29 and 4.56 μmol/g wet weight of NAA are hydrolysed at 6, 24, 48 and 120 hrs post‐sTBI, respectively. At the same time‐points increase in Asp is equal to 1.64, 2.57, 2.80 and 2.88 μmol/g wet weight, i.e. when taking into account Asp deriving from NAA hydrolysis deficits of 1.23, 1.55, 1.49 and 1.68 μmol/g wet weight of Asp are observed in the brain tissue of severely injured rats. It is highly probable that these amounts of Asp are rapidly converted into Glu *via* the activity of Asp aminotransferase, through the reaction Asp + α‐ketoglutarate ↔ Glu + oxaloacetate 11, thereby potentially linking degradation of NAA with Glu and its excitotoxic effects. As this phenomenon is confined to sTBI only, it can be postulaed that, under pathological conditions causing imbalance of NAA homeostasis with a net decrease in its cerebral concentration below a given threshold value (lower than the lowest value recorded in mTBI rats), an increase in Asp can trigger its transamination into Glu by aminotransferase, significantly concurring to cause an increase in Glu concentrations. To corroborate this hypothesis, it should be underlined that significantly lower values of Glu are present in the brain of rats affected by experimental Canavan disease 46, 47, the rare inborn error of metabolism caused by defects in the enzyme (*N*‐acetylaspartoacylase) degrading NAA into Asp and acetate 48. Under these pathological conditions, NAA is not hydrolysed by oligodendrocytes thus decreasing brain Asp availability, in turn producing about 50% lower Glu value 46, 47. That is, brain Glu metabolism is, at in lest in part, linked to the turnover rate of NAA. Therefore, control of NAA homeostasis at the enzymatic level might be a new, highly relevant target for new pharmacological approach aimed at reducing Glu excitotoxicity.

We found that GABA had transient increase at early stages post‐mTBI, and underwent significant increase in sTBI even at relatively long times after impact (24, 48, and 120 hrs), substantiating results of a very recent study obtained in a mouse model of mTBI 49. Since GABA is the only antagonist of Glu, it is conceivable that such an increase is aimed at counteracting the excitotoxic effect of increased Glu. However, if we take into account the Glu/GABA ratio, 7.65 is the value found in controls. Rats after mTBI exhibit a Glu/GABA ratio of 6.65, 5.67, 7.11, and 7.69 at 6, 24, 48 and 120 hrs post‐injury, respectively, whilst at the same time‐points sTBI animals have Glu/GABA ratios of 7.16, 7.29, 7.57, and 8.59. That is, the increase in GABA after sTBI was not sufficient to counteract excitotocity caused by the increase in Glu.

It is presumable that Glu decarboxylation, catalysed by Glu decarboxylase, would have the dual effect of decreasing total Glu concentration and simultaneously increasing GABA concentration. In light of this, it should be borne in mind that GABA is also part of a cycle between two cellular compartments: GABAergic neurons producing GABA from Glu and then releasing GABA to modulate excitatory effects of Glu, and astrocytes re‐uptaking GABA, deaminating and inserting it into the TCA cycle to indirectly generate first Glu and finally Gln which is again taken up by neurons, thereby closing the complex Glu‐Gln/GABA cycle occurring between neurons and astrocytes 11, 33. It has previously been demonstrated that the loss of one carbon unit occurring in neurons upon GABA formation, is restored in astrocytes using acetate as the preferential carbon donor substrate 11, 50. Again, it is therefore highly possible that acetate deriving from increased NAA hydrolysis, as it occurs in sTBI, may be used to support the increased rate of the Glu‐Gln/GABA cycle.

It is also possible that the increase in Tau in sTBI injured animals is correlated with the decrease in NAA and the concomitant decrease in osmolarity. It has been indicated that both compounds, besides various still debated biological functions 51, 52, 53, 54, 55, play a determining contribution in controlling cerebral osmolarity 52, 55, 56. Even though some studies showed a decrease in Tau after TBI 57, 58, 59, our results are in line with those reported by others 60, 61 and are logically connected to dysfunctional NAA homeostasis following sTBI and to a precise role of osmolite regulator performed by cerebral Tau. It is also possible that the increase in Tau is finalized to counteract Glu excitotoxic effects since it has been reported that Tau can directly interact with the NMDA receptor subtype decreasing the binding of Glu 62.

The tendency to modest, transient increase in excitatory amino acids in mTBI and to marked, protracted increase following sTBI is also clearly indicated by the changes in the brain values of free Ser and Gly, both interacting with the NMDA Glu receptors and actively contributing to the excitatory effects of Glu 63. Although we did not separate l‐Ser from d‐Ser (the stereoisomer actively binding to the NMDA receptors), it is presumable that the 1.3‐1.5 times increase in total Ser observed in sTBI rats generates an increase in the two isomers at least equal to the relative ratio recorded under physiological conditions, when d‐Ser has been reported to be about the 15% of the total Ser pool in adult rats 64. If this is correct and since the only source of cerebral d‐Ser is the racemization of l‐Ser *via* the d‐Ser racemase enzyme, a sufficient increase in the d‐Ser levels might have been formed after sTBI and contributed to Glu excitotoxicity. The considerable increase in Gly recorded during the first 48 hrs post‐sTBI completes the picture of a general exacerbation of excitatory phenomena, mediated by the increase in the aforementioned free amino acids that have been observed using different types of measures in various models of TBI 65, 66, 67.

In our experiments, we also found that sTBI, but not mTBI, causes significant changes in Arg and Citr concentration, and in the Arg/Citr ratio. This latter is an indirect indicator of the activity of NO synthases 68 and its decrease is generally attributed to the activation of iNOS, i.e. the inducible NOS isoenzyme responsible for cellular nitrosative stress 69. It is worth recalling that in previous experiments we found increased nitrosative stress following sTBI as evaluated by measuring the brain tissue concentration of nitrite and nitrate, the stable end‐products of NO metabolism 70.

The last remarkable finding of this study concerns a significant alteration of the concentrations of the amino acids and amino group containing compound involved in the so called methyl‐cycle, namely Met, SAH and l‐Cystat. Again, these changes were recorded only in brain tissue of animals experiencing a severe level of TBI (Table 1). Concomitant decrease in Met, and increase in SAH and l‐Cystatt strongly suggest either reduced availability of the main methyl‐group donor *S*‐adenosylmethionine (SAM) or a decreased rate in the formation of free cysteine (Cys) originating from the hydrolysis of l‐Cystat catalysed by cystathionine γ‐lyase. Alterations of SAM homeostasis have been proven to be involved in several neurodegenerative pathologies, including Alzheimer's disease 71 and multiple sclerosis 72. On the other hand, a Cys decrease may negatively influence the rate of GSH biosynthesis, thereby contributing to the previously observed decrease in GSH content as a consequence of marked oxidative stress occurring after sTBI 34. It was recently reported that groups of mTBI and sTBI patients had decreased levels of circulating Met and SAM, much more evident in sTBI than in mTBI patients, thus confirming the existence of an altered methyl‐cycle after a traumatic insult 73.

According to the timing of the changes in FAA, ACCG and NAA it is possible to evidence substantial differences between the two levels of injury. When considering mTBI, most of the changes in FAA, ACCG and NAA occurred at early‐phases post‐injury (6–24 hrs) and may therefore be attributed to the traumatic event and to the immediate onset of the neurometabolic cascade affecting various biochemical pathways 28, 67, At longer times post‐impact, animals experiencing mTBI showed normalization of the various compounds measured, indicating that reparative processes were mainly acting in the mildly injured brain after 48–120 hrs from trauma, as already found in previous studies using the same experimental model 27, 38, 45, 70. Concerning sTBI, it is possible to assume that the changes in FAA, ACCG and NAA occurring at early‐phases post‐injury (6–24 hrs) were also in this case related to the traumatic event and to the triggering of the neurometabolic cascade affecting various biochemical pathways 28, 67. At longer times post‐impact, rats receiving sTBI showed a further deterioration in the values of FAA, ACCG and NAA, very probably because of secondary mechanisms and reactions (inflammatory processes, oxidative/nitrosative stress, persistent mitochondrial malfunctioning) involving injured still viable cerebral tissue.

It is worth recalling that the results reported in the present study have been obtained using deproteinized brain samples (saved at −80°C) from three cohorts of rats (controls, mTBI and sTBI) in which, with the same methodology to induce TBI, we have previously shown that complex metabolic, enzymatic and gene mechanisms are responsible for profound changes in NAA homeostasis 35, sustained oxidative stress with neuroglobin overexpression and GSH depletion 34 and glucose dysmetabolism with energy metabolism imbalance 36. In these studies, differential injury severity caused transient (in mTBI rats) or irreversible changes in (in sTBI rats). By combining these previous data with those here shown and referring to FAA and ACCG, it is possible to conceptualize an even more detailed picture of the plethora of biochemical functions (and of their interrelationships) undergoing changes, the duration and severity of which only depend on the severity of injury.

In conclusion, although these results are not immediately translatable into the clinical setting particularly because of the differences between ‘rat time’ and ‘human time’, this study offers a panorama of the influence of different levels of TBI on the time course of changes of FAA and ACCG, highlighting previously unknown connections between alterations in NAA homeostasis and changes of amino acids directly or indirectly linked to post‐injury excitotoxic phenomena. These and other modifications (dysfunction of the methyl‐cycle, increased nitrosative stress) are clearly reversible and of limited magnitude in mTBI, thus corroborating the existence, and the rather complex biochemical basis, of a window of metabolic brain vulnerability after mTBI involving main cell biological functions 45, 67, 70. Given the number of the ‘disturbed’ biochemical tasks during this period of time, it is possible that repeat mTBIs may cause chronic impairment of these systems up to a point of permanent damages of key cellular bio‐molecular ‘apparatuses’. This might potentially be involved in the development of chronic post‐traumatic encephalopathy observed, for instance, in athletes with a long history of multiple concussions 74. Ultimately, we have again demonstrated that mTBI and sTBI have two clearly distinct patterns of molecular evolution, not in linear, proportional relationship. The force acting on the brain tissue at the time of the impact is the main determinant of the reactions ignited and, ultimately, of the fate of cerebral tissue. This should carefully be borne in mind when deciding the treatment of an mTBI or an sTBI patient and when developing new pharmacological interventions that could not be effective in all TBIs.

## Author contributions

AMA carried out the HPLC analysis of brain tissue, interpreted the analytical results and contributed to manuscript preparation. GL prepared brain tissue samples for the HPLC analyses, performed the analysis of amino acids and interpreted the chromatographic data. VDP performed the statistical analysis. SS carried out animal surgical procedures and revised the manuscript. GL wrote the first manuscript draft. AB contribute to perform statistical analysis and extensively revised the text. BT contributed to carry out animal surgical procedures, trauma induction, supervision of HPLC analyses and extensive manuscript revision.

## Conflict of interest

The authors confirm that there are no conflicts of interest.

## References

[1] Gundersen V , Storm‐Mathisen J , Bergersen LH . Neuroglial transmission. Physiol Rev. 2015; 95: 695–726. 26084688 10.1152/physrev.00024.2014

[2] Meunier CN , Dallérac G , Le Roux N , *et al* D‐serine and glycine differentially control neurotransmission during visual cortex critical period. PLoS ONE. 2016; doi:10.1371/journal.pone.0151233. 10.1371/journal.pone.0151233 PMC4803205 27003418

[3] Sekine A , Okamoto M , Kanatani Y , *et al* Amino acids inhibit kynurenic acid formation via suppression of kynurenine uptake or kynurenic acid synthesis in rat brain in vitro. Springerplus. 2015; doi:10.1186/s40064‐015‐0826‐9. 10.1186/s40064-015-0826-9 PMC4318830 25674503

[4] Olsen GM , Sonnewald U . Glutamate: Where does it come from and where does it go? Neurochem Int. 2015; 88: 47–52. 25447768 10.1016/j.neuint.2014.11.006

[5] Shnitko TA , Taylor SC , Stringfield SJ , *et al* Acute phenylalanine/tyrosine depletion of phasic dopamine in the rat brain. Psychopharmacology. 2016; 233: 2045–54. 26944052 10.1007/s00213-016-4259-0 PMC4864125

[6] De Simone R , Vissicchio F , Mingarelli C , *et al* Branched‐chain amino acids influence the immune properties of microglial cells and their responsiveness to pro‐inflammatory signals. Biochim Biophys Acta. 2013; 1832: 650–9. 23402925 10.1016/j.bbadis.2013.02.001

[7] Singhal NK , Li S , Arning E , *et al* Changes in methionine metabolism and histone h3 trimethylation are linked to mitochondrial defects in multiple sclerosis. J Neurosci. 2015; 35: 15170–86. 26558787 10.1523/JNEUROSCI.4349-14.2015 PMC6605362

[8] Gu F , Chauhan V , Chauhan A . Glutathione redox imbalance in brain disorders. Curr Opin Clin Nutr Metab Care. 2015; 18: 89–95. 25405315 10.1097/MCO.0000000000000134

[9] Cao Y , Gao Y , Xu S , *et al* Glutamate carboxypeptidase II gene knockout attenuates oxidative stress and cortical apoptosis after traumatic brain injury. BMC Neurosci. 2016; doi:10.1186/s12868‐016‐0251‐1. 10.1186/s12868-016-0251-1 PMC4836105 27091009

[10] Baslow MH . Evidence that the tri‐cellular metabolism of N‐acetylaspartate functions as the brain's “operating system”: how NAA metabolism supports meaningful intercellular frequency‐encoded communications. Amino Acids. 2010; 39: 1139–45. 20563610 10.1007/s00726-010-0656-6

[11] Cooper AJ , Jeitner TM . Central role of glutamate metabolism in the maintenance of nitrogen homeostasis in normal and hyperammonemic brain. Biomolecules. 2016; doi:10.3390/biom6020016. 10.3390/biom6020016 PMC4919911 27023624

[12] Singh AK , Ashraf M . Analysis of amino acids in brain and plasma samples by sensitive gas chromatography‐mass spectrometry. J Chromatogr. 1988; 425: 245–55. 3372639 10.1016/0378-4347(88)80029-x

[13] Wallwork JC , Sanstead AH . Effect of zinc deficiency on appetite and free amino acid concentrations in rat brain. J Nutr. 1983; 113: 47–54. 6822890 10.1093/jn/113.1.47

[14] Erecinska M , Nelson D , Wilson DF , *et al* Neurotransmitter amino acids in the CNS I. regional changes in amino acid levels in rat brain during ischemia and reperfusion. Brain Res. 1984; 304: 9–22. 6146383 10.1016/0006-8993(84)90857-6

[15] Bame M , Grier RE , Needleman R , *et al* Amino acids as biomarkers in the SOD1(G93A) mouse model of ALS. Biochim Biophys Acta. 2014; 1842: 79–87. 24129262 10.1016/j.bbadis.2013.10.004

[16] Bazán NG Jr . Changes in free fatty acids of brain by drug‐induced convulsions, electroshock and anaesthesia. J Neurochem. 1971; 18: 1379–85. 4328510 10.1111/j.1471-4159.1971.tb00002.x

[17] Patel HC , Bouamra O , Woodford M , *et al* Trends in head injury outcome from 1989 to 2003 and the effect of neurosurgical care: an observational study. Lancet. 2005; 366: 1538–44. 16257340 10.1016/S0140-6736(05)67626-X

[18] Hill CS , Coleman MP , Menon DK . Traumatic axonal injury: mechanisms and translational opportunities. Trends Neurosci. 2016; 39: 311–24. 27040729 10.1016/j.tins.2016.03.002 PMC5405046

[19] Reinert M , Hoelper B , Doppenberg E , *et al* Substrate delivery and ionic balance disturbance after severe human head injury. Acta Neurochir Suppl. 2000; 76: 439–44. 11450063 10.1007/978-3-7091-6346-7_91

[20] Balan IS , Saladino AJ , Aarabi B , *et al* Cellular alterations in human traumatic brain injury: changes in mitochondrial morphology reflect regional levels of injury severity. J Neurotrauma. 2013; 30: 367–81. 23131111 10.1089/neu.2012.2339 PMC3589878

[21] Vagnozzi R , Signoretti S , Floris R , *et al* Decrease in N‐acetylaspartate following concussion may be coupled to decrease in creatine. J Head Trauma Rehabil. 2013; 28: 284–92. 23249772 10.1097/HTR.0b013e3182795045

[22] Shin SS , Dixon CE . Alterations in cholinergic pathways and therapeutic strategies targeting cholinergic system after traumatic brain injury. J Neurotrauma. 2015; 32: 1429–40. 25646580 10.1089/neu.2014.3445 PMC4842943

[23] Titus DJ , Sakurai A , Kang Y , *et al* Phosphodiesterase inhibition rescues chronic cognitive deficits induced by traumatic brain injury. J Neurosci. 2013; 33: 5216–26. 23516287 10.1523/JNEUROSCI.5133-12.2013 PMC3655415

[24] Larner SF , Hayes RL , Wang KK . Unfolded protein response after neurotrauma. J Neurotrauma. 2006; 23: 807–29. 16774469 10.1089/neu.2006.23.807

[25] Di Pietro V , Amin D , Pernagallo S , *et al* Transcriptomics of traumatic brain injury: gene expression and molecular pathways of different grades of insult in a rat organotypic hippocampal culture model. J Neurotrauma. 2010; 27: 349–59. 19903084 10.1089/neu.2009.1095

[26] Hui H , Rao W , Zhang L , *et al* Inhibition of Na(+)‐K(+)‐2Cl(‐) Cotransporter‐1 attenuates traumatic brain injury‐induced neuronal apoptosis via regulation of Erk signaling. Neurochem Int. 2016; 94: 23–31. 26854573 10.1016/j.neuint.2016.02.002

[27] Tavazzi B , Signoretti S , Lazzarino G , *et al* Cerebral oxidative stress and depression of energy metabolism correlate with severity of diffuse brain injury in rats. Neurosurgery. 2005; 56: 582–9. 15730584 10.1227/01.neu.0000156715.04900.e6

[28] Signoretti S , Vagnozzi R , Tavazzi B , *et al* Biochemical and neurochemical sequelae following mild traumatic brain injury: summary of experimental data and clinical implications. Neurosurg Focus. 2010; doi:10.3171/2010.9.FOCUS10183. 10.3171/2010.9.FOCUS10183 21039135

[29] Kinoshita K . Traumatic brain injury: pathophysiology for neurocritical care. J Intensive Care. 2016; doi:10.1186/s40560‐016‐0138‐3. 10.1186/s40560-016-0138-3 PMC4847183 27123305

[30] Chen X , Zhao Z , Chai Y , *et al* The incidence of critical‐illness‐related‐corticosteroid‐insufficiency is associated with severity of traumatic brain injury in adult rats. J Neurol Sci. 2014; 342: 93–100. 24819916 10.1016/j.jns.2014.04.032

[31] Bartnik‐Olson BL , Oyoyo U , Hovda DA , *et al* Astrocyte oxidative metabolism and metabolite trafficking after fluid percussion brain injury in adult rats. J Neurotrauma. 2010; 27: 2191–202. 20939699 10.1089/neu.2010.1508 PMC2996847

[32] Chamoun R , Suki D , Gopinath SP , *et al* Role of extracellular glutamate measured by cerebral microdialysis in severe traumatic brain injury. J Neurosurg. 2010; 113: 564–70. 20113156 10.3171/2009.12.JNS09689 PMC3464461

[33] Guerriero RM , Giza CC , Rotenberg A . Glutamate and GABA imbalance following traumatic brain injury. Curr Neurol Neurosci Rep. 2015; doi:10.1007/s11910‐015‐0545‐1. 10.1007/s11910-015-0545-1 PMC4640931 25796572

[34] Di Pietro V , Lazzarino G , Amorini AM , *et al* Neuroglobin expression and oxidant/antioxidant balance after graded traumatic brain injury in the rat. Free Radic Biol Med. 2014; 69: 258–64. 24491879 10.1016/j.freeradbiomed.2014.01.032

[35] Di Pietro V , Amorini AM , Tavazzi B , *et al* The molecular mechanisms affecting N‐acetylaspartate homeostasis following experimental graded traumatic brain injury. Mol Med. 2014; 20: 147–57. 24515258 10.2119/molmed.2013.00153 PMC3966992

[36] Amorini AM , Lazzarino G , Di Pietro V , *et al* Metabolic, enzymatic and gene involvement in cerebral glucose dysmetabolism after traumatic brain injury. Biochim Biophys Acta. 2016; 1862: 679–87. 26844378 10.1016/j.bbadis.2016.01.023

[37] Marmarou A , Foda MA , van den Brink W , *et al* A new model of diffuse brain injury in rats. Part I: Pathophysiology and biomechanics. J Neurosurg. 1994; 80: 291–300. 8283269 10.3171/jns.1994.80.2.0291

[38] Signoretti S , Marmarou A , Tavazzi B , *et al* N‐Acetylaspartate reduction as a measure of injury severity and mitochondrial dysfunction following diffuse traumatic brain injury. J Neurotrauma. 2001; 18: 977–91. 11686498 10.1089/08977150152693683

[39] Lazzarino G , Nuutinen M , Tavazzi B , *et al* A method for preparing freeze‐clamped tissue samples for metabolite analyses. Anal Biochem. 1989; 181: 239–41. 2817387 10.1016/0003-2697(89)90236-4

[40] Lazzarino G , Amorini AM , Fazzina G , *et al* Single‐sample preparation for simultaneous cellular redox and energy state determination. Anal Biochem. 2003; 322: 51–9. 14705780 10.1016/j.ab.2003.07.013

[41] Amorini AM , Giorlandino C , Longo S , *et al* Metabolic profile of amniotic fluid as a biochemical tool to screen for inborn errors of metabolism and fetal anomalies. Mol Cell Biochem. 2012; 359: 205–16. 21837404 10.1007/s11010-011-1015-y

[42] Tavazzi B , Lazzarino G , Leone P , *et al* Simultaneous high performance liquid chromatographic separation of purines, pyrimidines, N‐acetylated amino acids, and dicarboxylic acids for the chemical diagnosis of inborn errors of metabolism. Clin Biochem. 2005; 38: 997–1008. 16139832 10.1016/j.clinbiochem.2005.08.002

[43] Purins K , Lewén A , Hillered L , *et al* Brain tissue oxygenation and cerebral metabolic patterns in focal and diffuse traumatic brain injury. Front Neurol. 2014; doi:10.3389/fneur.2014.00064. 10.3389/fneur.2014.00064 PMC4013462 24817863

[44] Bouzat P , Sala N , Suys T , *et al* Cerebral metabolic effects of exogenous lactate supplementation on the injured human brain. Intensive Care Med. 2014; 40: 412–21. 24477453 10.1007/s00134-013-3203-6

[45] Vagnozzi R , Tavazzi B , Signoretti S , *et al* Temporal window of metabolic brain vulnerability to concussions: mitochondrial‐related impairment–part I. Neurosurgery. 2007; 61: 379–88. 10.1227/01.NEU.0000280002.41696.D8 17762751

[46] Surendran S , Rady PL , Michals‐Matalon K , *et al* Expression of glutamate transporter, GABRA6, serine proteinase inhibitor 2 and low levels of glutamate and GABA in the brain of knock‐out mouse for Canavan disease. Brain Res Bull. 2003; 61: 427–35. 12909286 10.1016/s0361-9230(03)00158-8

[47] Surendran S , Matalon KM , Szucs S , *et al* Metabolic changes in the knockout mouse for Canavan's disease: implications for patients with Canavan's disease. J Child Neurol. 2003; 18: 611–5. 14572139 10.1177/08830738030180090701

[48] Baslow MH , Guilfoyle DN . Canavan disease, a rare early‐onset human spongiform leukodystrophy: insights into its genesis and possible clinical interventions. Biochimie. 2013; 95: 946–56. 23151389 10.1016/j.biochi.2012.10.023

[49] Schneider BL , Ghoddoussi F , Charlton JL , *et al* Increased cortical gamma‐aminobutyric acid precedes incomplete extinction of conditioned fear and increased hippocampal excitatory tone in a mouse model of mild traumatic brain injury. J Neurotrauma. 2016; 33: 1614–24. 26529240 10.1089/neu.2015.4190

[50] Rae C , Fekete AD , Kashem MA , *et al* Metabolism, compartmentation, transport and production of acetate in the cortical brain tissue slice. Neurochem Res. 2012; 37: 2541–53. 22851350 10.1007/s11064-012-0847-5

[51] Vitvitsky V , Garg SK , Banerjee R . Taurine biosynthesis by neurons and astrocytes. J Biol Chem. 2011; 286: 32002–10. 21778230 10.1074/jbc.M111.253344 PMC3173217

[52] Choe KY , Olson JE , Bourque CW . Taurine release by astrocytes modulates osmosensitive glycine receptor tone and excitability in the adult supraoptic nucleus. J Neurosci. 2012; 32: 12518–27. 22956842 10.1523/JNEUROSCI.1380-12.2012 PMC6621246

[53] Madhavarao CN , Arun P , Moffett JR , *et al* Defective N‐acetylaspartate catabolism reduces brain acetate levels and myelin lipid synthesis in Canavan's disease. Proc Natl Acad Sci U S A. 2005; 102: 5221–6. 15784740 10.1073/pnas.0409184102 PMC555036

[54] Clark JF , Doepke A , Filosa JA , *et al* N‐acetylaspartate as a reservoir for glutamate. Med Hypotheses. 2006; 67: 506–12. 16730130 10.1016/j.mehy.2006.02.047

[55] Baslow MH . Evidence supporting a role for N‐acetyl‐L‐aspartate as a molecular water pump in myelinated neurons in the central nervous system. An analytical review. Neurochem Int. 2002; 40: 295–300. 11792458 10.1016/s0197-0186(01)00095-x

[56] Oenarto J , Görg B , Moos M , *et al* Expression of organic osmolyte transporters in cultured rat astrocytes and rat and human cerebral cortex. Arch Biochem Biophys. 2014; 560: 59–72. 25004465 10.1016/j.abb.2014.06.024

[57] Stover JF , Morganti‐Kosmann MC , Lenzlinger PM , *et al* Glutamate and taurine are increased in ventricular cerebrospinal fluid of severely brain‐injured patients. J Neurotrauma. 1999; 16: 135–42. 10098958 10.1089/neu.1999.16.135

[58] Stover JF , Unterberg AW . Increased cerebrospinal fluid glutamate and taurine concentrations are associated with traumatic brain edema formation in rats. Brain Res. 2000; 875: 51–5. 10967298 10.1016/s0006-8993(00)02597-x

[59] Seki Y , Kimura M , Mizutani N , *et al* Cerebrospinal fluid taurine after traumatic brain injury. Neurochem Res. 2005; 30: 123–8. 15756940 10.1007/s11064-004-9693-4

[60] Pascual JM , Solivera J , Prieto R , *et al* Time course of early metabolic changes following diffuse traumatic brain injury in rats as detected by (1)H NMR spectroscopy. J Neurotrauma. 2007; 24: 944–59. 17600512 10.1089/neu.2006.0190

[61] Zhuo J , Keledjian K , Xu S , *et al* Changes in diffusion kurtosis imaging and magnetic resonance spectroscopy in a direct cranial blast traumatic brain injury (dc‐bTBI) model. PLoS ONE. 2015; doi:10.1371/journal.pone.0136151. 10.1371/journal.pone.0136151 PMC4547765 26301778

[62] Chan CY , Sun HS , Shah SM , *et al* Direct interaction of taurine with the NMDA glutamate receptor subtype via multiple mechanisms. Adv Exp Med Biol. 2013; 775: 45–52. 23392923 10.1007/978-1-4614-6130-2_4

[63] Pinto MC , Lima IV , da Costa FL , *et al* Glycine transporters type 1 inhibitor promotes brain preconditioning against NMDA‐induced excitotoxicity. Neuropharmacology. 2015; 89: 274–81. 25312280 10.1016/j.neuropharm.2014.10.003

[64] Song Y , Feng Y , Lu X , *et al* D‐Amino acids in rat brain measured by liquid chromatography/tandem mass spectrometry. Neurosci Lett. 2008; 445: 53–57. 18775473 10.1016/j.neulet.2008.08.058 PMC2585614

[65] Pu B , Xue Y , Wang Q , *et al* Dextromethorphan provides neuroprotection via anti‐inflammatory and anti‐excitotoxicity effects in the cortex following traumatic brain injury. Mol Med Rep. 2015; 12: 3704–10. 26004757 10.3892/mmr.2015.3830

[66] Quintard H , Patet C , Suys T , *et al* Normobaric hyperoxia is associated with increased cerebral excitotoxicity after severe traumatic brain injury. Neurocrit Care. 2015; 22: 243–50. 25168744 10.1007/s12028-014-0062-0

[67] Giza CC , Hovda DA . The new neurometabolic cascade of concussion. Neurosurgery. 2014; 75(Suppl 4): S24–33. 10.1227/NEU.0000000000000505 PMC4479139 25232881

[68] Pérez‐Neri I , Castro E , Montes S , *et al* Arginine, citrulline and nitrate concentrations in the cerebrospinal fluid from patients with acute hydrocephalus. J Chromatogr B Analyt Technol Biomed Life Sci. 2007; 851: 250–6. 10.1016/j.jchromb.2006.10.047 17110176

[69] Cai X , Li X , Li L , *et al* Adiponectin reduces carotid atherosclerotic plaque formation in ApoE‐/‐ mice: roles of oxidative and nitrosative stress and inducible nitric oxide synthase. Mol Med Rep. 2015; 11: 1715–21. 25395016 10.3892/mmr.2014.2947 PMC4270320

[70] Tavazzi B , Vagnozzi R , Signoretti S , *et al* Temporal window of metabolic brain vulnerability to concussions: oxidative and nitrosative stresses–part II. Neurosurgery. 2007; 61: 390–5. 10.1227/01.neu.0000255525.34956.3f 17806141

[71] Panza F , Frisardi V , Capurso C , *et al* Possible role of S‐adenosylmethionine, S‐adenosylhomocysteine, and polyunsaturated fatty acids in predementia syndromes and Alzheimer's disease. J Alzheimers Dis. 2009; 16: 467–70. 19276539 10.3233/JAD-2009-1012

[72] Suchy J , Lee S , Ahmed A , *et al* Dietary supplementation with S‐adenosyl methionine delays the onset of motor neuron pathology in a murine model of amyotrophic lateral sclerosis. Neuromolecular Med. 2010; 12: 86–97. 19757209 10.1007/s12017-009-8089-7

[73] Dash PK , Hergenroeder GW , Jeter CB , *et al* Traumatic brain injury alters methionine metabolism: implications for pathophysiology. Front Syst Neurosci. 2016; doi:10.3389/fnsys.2016.00036. 10.3389/fnsys.2016.00036 PMC4850826 27199685

[74] Omalu B . Chronic traumatic encephalopathy. Prog Neurol Surg. 2014; 28: 38–49. 24923391 10.1159/000358761

